# Supplementary material for: What slows the progress of health systems strengthening at subnational level? A political economy analysis of three districts in Uganda
Source: PLOS Glob Public Health. 2025 Jul 8;5(7):e0002673. doi: 10.1371/journal.pgph.0002673 (PMC12237036; doi:10.1371/journal.pgph.0002673)
Supplement: Attachment — Submitted filename: PLOS- political Economy Analysis.docx [file pgph.0002673.s003.docx]

**Summary:** This study was based on the topic “What slows the progress of health systems strengthening at subnational level? A political economy analysis of three districts in Uganda”. The subject matter is important and the authors should be applauded on their choice of topic. 

There are a few major and some minor revisions present in the current submission that need to be addressed. Since this is a very important topic, I would like to strongly encourage the authors to consider addressing the issues raised to strengthen the work for consideration.

**Key Message**

Minor Revisions

Line 64: It should be ‘the challenges of’ instead of ‘the challenges for’

**Body of Manuscript**

**Introduction**

Minor Essential Revisions
Line 91: ‘Yet, the goal for UHC’ should be ‘Yet, the goal of UHC’

Line 114: ‘health system blocks’ should be ‘health systems building blocks’

Line 122-123: Delete ‘in Uganda’ at the end of the sentence. It’s a repetition

Line 141: Delete ‘do’ in ‘a) how do the financing….’

**Materials and Methods**
Minor Essential Revisions
Line 147: ‘The data used in this paper are drawn...’ should be ‘the data used in this paper were drawn…’

Line 163: What do the authors mean by a multiple case study design? What makes the design for this study a multiple case study?

Line 171: Table 1 should be reformatted into scientific / publication format

**Data collection**

Major Compulsory Revisions

Authors should provide a description of the problem-driven PEA conceptual framework

Line 178: PEA should be written in full first

Line 183-184: Authors should reformat Table 2 into scientific / publication format

Line 188: MOH should be written in full first

Authors should provide description of the qualitative data that was collected. What data were collected? In other words, what did the interview guide entail?

Authors should provide description of how the data from the document review were collected. How were the documents identified? What specific data were collected and what tool was used to chart the data?

**Data analysis**
Minor Essential Revisions

Authors should provide a brief description on how the data from the document review were analysed?

**Results**
Minor Essential Revisions

Line 247: ‘Insufficient” should be ‘insufficient’

Line 250: What resources. I think the authors should state it clearly in the subheading if its ‘insufficient financial and non-financial resources’ or just ‘insufficient financial resources’

Line 259: replace ‘our’ with ‘the’

Line 266: ‘year established’ should be ‘year of establishment’

Line 267: What do the authors mean by ‘the board’. Also, what do the authors mean by ‘centre’ in the sentence ‘moreover, the main source of funding from the centre…’

Line 275: Should be ‘findings from the document review…’

Line 287: Delays in disbursement of what?

Line 323: Authors should reformat the table 3

Line 354: ‘Resource allocation priorities’ should be rephrased e.g ‘misplaced resource allocation priorities’ or ‘biased resource allocation priorities’ etc

Line 365: use the abbreviated version of implementing partners

Line 378: ‘the respondents attributed blamed this on inadequacy of resources’ doesn’t read well

Line 403: ‘with in’ should be ‘within’

Line 430: ‘…accountability on top of those from…’ Do you mean in addition to? On top of those doesn’t read well

Line 462: ‘donors should to address some’ doesn’t read well

Line 564: ‘varied’ should be ‘vary’

**Discussions**

Minor Essential Revisions

Line 612: What do the authors mean by ‘to stretch health dollars’

Line 627: Should be ‘study strengths and limitations’

**Conclusion**

Minor Essential Revisions

Line 650: who are ‘vote holders’ or what does it mean

Line 657-659: I don’t think this sentence ‘Development partners will continue to play a significant role in compensation for financial and technical local shortfalls’ is necessary. The authors should be straightforward with the conclusion.
